# Actin Alpha 2 Downregulation Inhibits Neural Stem Cell Proliferation and Differentiation into Neurons through Canonical Wnt/*β*-Catenin Signaling Pathway

**DOI:** 10.1155/2022/7486726

**Published:** 2022-02-09

**Authors:** Ji Zhang, Quan Hu, Xuheng Jiang, Shuhong Wang, Xin Zhou, Yuanlan Lu, Xiaofei Huang, Haizhen Duan, Tianxi Zhang, Hongfei Ge, Anyong Yu

**Affiliations:** Department of Emergency, Affiliated Hospital of Zunyi Medical University, 563003 Zunyi, Guizhou, China

## Abstract

Our previous study has shown that actin alpha 2 (ACTA2) is expressed in NSC and ACTA2 downregulation inhibits NSC migration by increasing RhoA expression and decreasing the expression of Rac1 to curb actin filament polymerization. Given that proliferation and differentiation are the two main characteristics of NSC, the role of ACTA2 downregulation in the proliferation and differentiation of NSC remains elusive. Here, the results demonstrated that ACTA2 downregulation using ACTA2 siRNA held the potential of inhibiting NSC proliferation using cell counting kit-8 (CCK8) and immunostaining. Then, our data illustrated that ACTA2 downregulation attenuated NSC differentiation into neurons, while directing NSC into astrocytes and oligodendrocytes using immunostaining and immunoblotting. Thereafter, the results revealed that the canonical Wnt/*β*-catenin pathway was involved in the effect of ACTA2 downregulation on the proliferation and differentiation of NSC through upregulating p-*β*-catenin and decreasing *β*-catenin due to inactivating GSK-3*β*, while this effect could be partially abolished with administration of CHIR99012, a GSK-3 inhibitor. Collectively, these results indicate that ACTA2 downregulation inhibits NSC proliferation and differentiation into neurons through inactivation of the canonical Wnt/*β*-catenin pathway. The aim of the present study is to elucidate the role of ACTA2 in proliferation and differentiation of NSC and to provide an intervention target for promoting NSC proliferation and properly directing NSC differentiation.

## 1. Introduction

Neural stem cells (NSC), a subtype of undifferentiated and multipotent neural cells, are available in the central nervous system (CNS) to generate three main lineages of neural cells including neurons, astrocytes, and oligodendrocytes [1–3]. Previous studies have illustrated that the subventricular zone (SVZ) of the lateral ventricle and the subgranular zone (SGZ) of the hippocampus are two neurogenic regions harboring NSC in the adult mammal brains [4, 5]. Meanwhile, the adult NSC are proliferated in SVZ and migrated towards lesions to exert multiple neurorestorative effects after brain injury [4]. These neurorestorative effects including but not limited to cell replacement, neuromodulation, and neurotrophic support to accelerate functional recovery in various CNS diseases such as stroke [4, 6], traumatic brain injury (TBI) [7], spinal cord injury (SCI) [7, 8], neurodegenerative diseases [6, 9], neuropsychiatric disorders [10], and others [11, 12]. However, the vast majority of NSC originated from SVZ differentiate into astrocytes to generate glial scars after central neuropathy [13–15]. Consequently, exploring approaches to direct NSC differentiation into neurons, instead of astrocytes, might be a viable avenue to broaden the application of NSC for the treatment of CNS insults.

Actin alpha 2 (ACTA2), also called alpha smooth muscle actin (*α*-SMA), might be a candidate to regulate the proliferation and differentiation of NSC. Our previous investigation shows that ACTA2 is expressed in NSC and ACTA2 downregulation inhibits NSC migration by increasing RhoA expression and decreasing the expression of Rac1 to curb actin filament polymerization [16]. Meanwhile, studies reflect that reduction in the formation of actin filament polymerization resulting from gene silencing of profilin-1 suppresses endothelial proliferation [17, 18]. Furthermore, recent research also demonstrates that the proliferative capacity is clearly diminished when the expression of ACTA2 is downregulated in myofibroblasts [19], indicating that ACTA2 might be a mediator in regulating NSC proliferation. Coincidently, a recent study demonstrates that valproic acid (VPA) holds the ability of promoting hematopoietic stem cell (HSC) into megakaryocyte and thrombocyte through modulating actin filament polymerization [20]. In addition, a report has illustrated that cyclase-associated protein 2 (VAP2), a family of actin regulators, plays a pivotal role in myofibril differentiation via regulating actin filament polymerization [21], implying that ACTA2 might be involved in regulating NSC differentiation. Therefore, identifying the role of ACTA2 in the proliferation and differentiation of NSC and deciphering the underlying mechanism are worthy of probing.

The canonical (*β*-catenin-dependent) Wnt signaling pathway elicits pleiotropic effects of NSC in diverse cellular and physiological processes including asymmetric cell division, proliferation, expansion, differentiation, and maturation during embryonic development and tissue rehabilitation [15, 22–24]. In the canonical Wnt signaling pathway, *β*-catenin accumulation is negatively regulated by a protein compound that involves glycogen synthase kinase-3 (GSK-3), adenomatous polyposis coli (APC), casein kinase 1 (CK1), and Axin [22]. This degradation complex is disrupted when Wnt protein binds to receptors of the frizzled (FZD) and low-density lipoprotein receptor-related protein (LRP5/6) families [22]. Our previous study substantiates that ambroxol facilitates NSC differentiation into neurons and suppressing NSC direction into astrocytes through decreasing GSK-3 activity, thereafter increasing *β*-catenin deposition in penumbra after ischemic stroke [15], implying that the activity of GSK-3 might be associated with NSC differentiation induced by ACTA2.

In the present study, we posited that ACTA2 took part in regulating the proliferation and differentiation of NSC, and the underlying mechanism was negatively correlated with the activity of GSK-3. Firstly, the primary NSC were cultured. Then, the effect of ACTA2 downregulation on the expansion and differentiation of NSC was evaluated using cell counting kit-8 (CCK8), immunostaining, and immunoblotting. Subsequently, the role of the canonical Wnt/*β*-catenin signaling pathway in ACTA2 mediating proliferation and differentiation of NSC was determined using immunostaining and immunoblotting with the addition of GSK-3 antagonist. The aim of the current study is to elucidate the role of ACTA2 in the proliferation and differentiation of NSC and its underlying mechanism(s) and provide an intervention target for properly directing NSC differentiation, thereafter attenuating the pathological deterioration and facilitating functional recovery after central neuropathy, even in peripheral neuropathy.

## 2. Materials and Methods

### 2.1. Animals

Embryonic *C57BL/6* mice were purchased from Animal Experimental Center of Zunyi Medical University. This work was approved by the local authorities of Zunyi Medical University for the laboratory use of animals (approve no. KLLY(A)-2020-012) and supervised by the Ethics Committee of Affiliated Hospital of Zunyi Medical University. All experimental procedures were performed according to China's animal welfare legislation for the protection of animals used for scientific purposes.

### 2.2. Primary NSC Culture

Embryos on day 14.5 from 28 pregnant C57BL/6 mice were used to culture primary NSC, as previously described [15, 16]. In brief, the neonatal cerebral cortices were dissected and twice rinsed with Dulbecco's Modified Eagle's Medium (DMEM, Boster, Wuhan, China). Thereafter, tissues were immersed in 0.125% trypsin-EDTA (Gibco, Grand Island, NY, USA) at 37°C for 30 minutes. Then, the samples were twice rinsed with DMEM after being washed with 10% fetal bovine serum (FBS; Hyclone, Logan, Utah, USA) for three times to eliminate the activity of trypsin. Next, the samples were triturated using a fire-polished Pasteur pipette and percolated through a 70 *μ*m Nylon cell strainer (Nest, Wuxi, Jiangsu, China) after they were rinsed twice with DMEM. Subsequently, cell suspensions were cultured in enrichment medium-DMEM/F12 (Boster, Wuhan, China) supplemented with 2% B27 (Gibco, Grand Island, NY, USA), 20 ng/ml recombinant murine epidermal growth factor (EGF; Peprotech, Rocky Hill, NJ, USA), and 20 ng/ml recombinant murine fibroblast growth factor-basic (bFGF; Peprotech, Rocky Hill, NJ, USA) at 37°C under 5% CO_2_ humidified condition. NSC was allowed to grow in a floating situation to form neurospheres.

For NSC passage, neurospheres were firstly centrifuged at the speed of 300 rpm. Then, neurospheres were dissociated in StemPro Accutase Cell Dissociation Reagent (Gibco, Grand Island, NY, USA) for 12 minutes at room temperature. After being rinsed twice using enrichment medium, the single cell suspensions were grown in the enrichment medium. The passage of NSC used for all experiments in this study was from 3 to 5.

CHIR99012 was purchased from Sigma-Aldrich (St. Louis, MO, USA) and firstly dissolved in dimethyl sulfoxide (DMSO; St. Louis, MO, USA) at a storage concentration of 3 mM. Then, CHIR99012 was diluted in culture medium at a working concentration of 3 *μ*M, as previously reported [25].

### 2.3. Cell Counting Kit-8 (CCK8)

Cell counting kit-8 (CCK8; Dojindo, Tokyo, Japan) was applied to evaluate the cell proliferation index reflected the cell viability. Briefly, a total of 100 *μ*l of single cell suspension (~10000 cells/well) was placed in a 96-well cell culture cluster precoated with 10 *μ*g/ml poly-L-ornithine (PLO; Sigma-Aldrich, St. Louis, MO, USA). Then, 10 *μ*l CCK8 solution was added in each well and incubated for 2.5 hours at 37°C. Afterwards, the absorbance value was determined at a test wavelength of 450 nm using a microplate reader (Varioskan, Thermo Fisher Scientific, Waltham, MA), and 630 nm was used as a reference wavelength as well.

### 2.4. NSC Differentiation

For differentiation, NSC were plated on 10 *μ*g/ml PLO precoated petri dishes or coverslips. Then, they were incubated in differentiation medium-DMEM/F12 supplemented with B27 (Gibco, Grand Island, NY, USA) and 1% Glutamax (Gibco, Grand Island, NY, USA) for 10 days as previously reported [15].

### 2.5. Immunofluorescence

NSC plated on PLO precoated coverslips were firstly fixed using 4% paraformaldehyde (PFA) for 10 min at room temperature. Afterwards, the samples were rinsed with phosphate buffer saline (PBS, pH ~7.4) for three times. Thereafter, the coverslips were blocked by 5% bovine serum albumin (BSA; Beyotime, Shanghai, China) for 2 hours after being penetrated with 0.25% Triton X-100 in PBS for 30 minutes. Subsequently, the specimens were incubated with primary antibodies as follows: mouse anti-Olig2 (Millipore, Billerica, MA, USA), rabbit anti-*β*-III-Tubulin (1 : 100, Cell Signaling Technology, Danvers, MA, USA), rabbit anti-glial fibrillary acidic protein (GFAP; 1 : 100, Abcam, Cambridge, UK), rabbit anti-Ki-67 (1 : 100, Abcam, Cambridge, UK), goat anti-Nestin (1 : 100, Santa Cruz Biotechnology, CA, USA), rabbit anti-Sox2 (1 : 100, Cell Signaling Technology, Danvers, MA, USA), rabbit anti-MAP2 (1 : 100, Abcam, Cambridge, UK), and rabbit anti-non-phospho (active) *β*-catenin (1 : 100, Cell Signaling Technology, Danvers, MA) overnight at 4°C. On the next day, the relative fluorescence secondary antibodies were incubated at room temperature for 2 hours after washing with PBS for three times. Finally, cell nuclei were counterstained with 4′-6-Diamidino-2-phenylindole (DAPI, Sigma-Aldrich, St. Louis, MO, USA) for 10 minutes at room temperature. Then, coverslips were mounted onto glass slides, and the immunostaining images were visualized with a confocal microscope (Carl Zeiss, LSM780, Weimar, Germany) and analyzed by a Zen 2011 software (Carl Zeiss, Weimar, Germany).

### 2.6. ACTA2 siRNA Transfection

ACTA2-specific siRNA (sc-43591) was purchased from Santa Cruz Biotechnology (CA, USA). ACTA2-specific siRNA was transfected into NSC using a Lipofectamine™ 3000 transfection reagent (Invitrogen, Waltham, MA, USA) according to the manufacturer's instructions. Meanwhile, the same volume of scramble siRNA and Lipofectamine™ 3000 transfection reagent was loaded as negative control. The transfection efficiency was determined by immunoblotting, as previously described (data not shown) [16].

### 2.7. Immunoblotting

Samples in each group were homogenized with RIPA lysis and extraction buffer (Thermo Fisher Scientific, Waltham, MA, USA) supplemented with protease and phosphatase inhibitors (Roche, Indianapolis, IN, USA). Then, the specimens were placed on ice for 30 minutes. The supernatant from each sample was collected by being centrifuged at 12000 rpm for 30 minutes at 4°C. The protein concentration was examined by an enhanced bicinchoninic acid (BCA) Protein Assay Kit (Beyotime, Shanghai, China). Thereafter, equal quality of protein was separated by 10% sodium dodecyl sulfate-polyacrylamide gel electrophoresis (SDS-PAGE) under reducing conditions and electro-blotted to polyvinylidene difluoride (PVDF) membranes (Beyotime, Shanghai, China). Subsequently, the membranes were blocked in 5% (*w*/*v*) nonfat dry milk (Boster, Wuhan, China) that was dissolved in 0.5% Tween-20 in Tris-buffered saline (TBST) at room temperature for 2 hours. Afterward, the membranes were immersed in primary antibodies, mouse anti-GADPH (Santa Cruz Biotechnology, CA, USA), mouse anti-Olig2 (Millipore, Billerica, MA, USA), rabbit anti-*β*-III-Tubulin (Cell Signaling Technology, Danvers, MA, USA), rabbit anti-glial fibrillary acidic protein (GFAP; Abcam, Cambridge, UK), rabbit anti-non-phospho (active) *β*-catenin (Cell Signaling Technology, Danvers, MA, USA), rabbit anti-phospho-*β*-catenin (Cell Signaling Technology, Danvers, MA, USA), mouse anti-GSK-3*β* (Cell Signaling Technology, Danvers, MA, USA), and rabbit anti-phospho-GSK-3*β* (Cell Signaling Technology, Danvers, MA, USA) overnight at 4°C. Afterwards, the membranes were incubated with the corresponding horseradish peroxidase- (HRP-) conjugated secondary antibody (Boster, Wuhan, China) at room temperature for 2 hours after being washed with TBST for 3 times. Finally, the bands were visualized by a ChemiDoc™ XRS^+^ imaging system (Bio-Rad, California, USA) using a WesternBright ECL Kit (Thermo Fisher Scientific, Waltham, MA, USA). The optical density of each membrane was measured using an Image Lab™ software (Bio-Rad, California, USA). GAPDH was served as a loading control.

### 2.8. Statistical Analysis

All data were expressed as the mean ± SD and analyzed using a SPSS 20.0 software (SPSS, Inc., Chicago, IL, USA). The normal distribution of all data in each group was tested using a Shapiro–Wilk normality test. If the data were normal distribution, the statistical analysis was conducted using one-way analysis of variance (ANOVA), followed by Turkey's post hoc test among multiple comparisons. Comparison between the two groups was determined by Student's *t*-test. Meanwhile, data were shown as median and interquartile range (IQR) and analyzed using the Mann–Whitney *U* test if the data were failed the normality test. Statistical significance was defined as ^∗^*p* < 0.05,  ^∗∗^*p* < 0.01, or ^∗∗∗^*p* < 0.001.

## 3. Results

### 3.1. Primary Mouse NSC Culture and Characteristics

**Figure 1:**
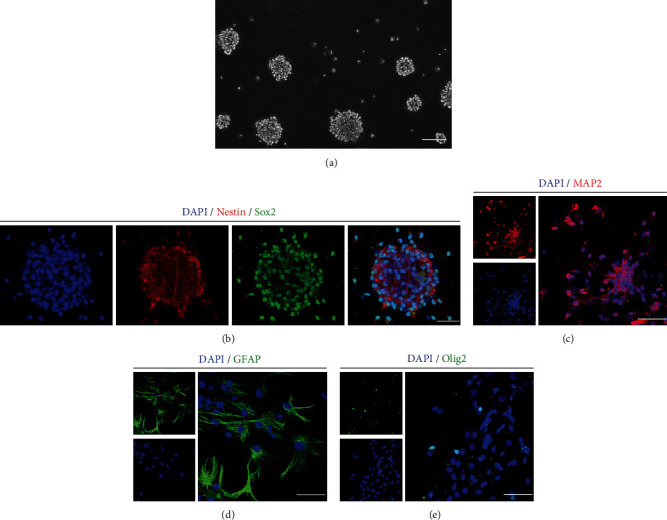
Primary NSC culture and characteristics. (a) The suspended neurospheres were notably floating in enrichment medium on day 3. Scale bar: 100 *μ*m. (b) Immunostaining images indicating most of cultured cells expressed Nestin and Sox2. Cell nuclei were counterstained with DAPI in blue. Scale bar: 20 *μ*m. (c) Immunostaining images showing cultured cells bore the multipotency of differentiation into MAP2^+^ cells. Cell nuclei were counterstained with DAPI in blue. Scale bars: 10 *μ*m. (d) Immunostaining images demonstrating cultured cells held the ability of differentiation into GFAP^+^ cells. Cell nuclei were counterstained with DAPI in blue. Scale bars: 10 *μ*m. (e) Immunostaining images delineating cultured cells possessed the capacity of differentiation into Olig2^+^ cells. Cell nuclei were counterstained with DAPI in blue. Scale bar: 10 *μ*m.

For primary mouse NSC culture, cell suspensions were grown in enrichment medium, and neurospheres were clearly observed after 3 days (Figure 1(a)). Furthermore, the majority of cultured cells in neurosphere were immunopositive for Nestin and Sox2, two of the NSC markers [26, 27], using immunostaining assays (Figure 1(b)). Thereafter, to determine the multipotency of cultured cells, cells were incubated in differentiation medium for 10 days. The immunostaining images depicted the cultured cells bore the ability of differentiation into neurons (MAP2^+^) (Figure 1(c)), astrocytes (GFAP^+^) (Figure 1(d)), and oligodendrocytes (Olig2^+^) (Figure 1(e)). Collectively, these results elucidated that the cultured cells were NSC and used for future research.

### 3.2. ACTA2 Downregulation Inhibited NSC Proliferation

**Figure 2:**
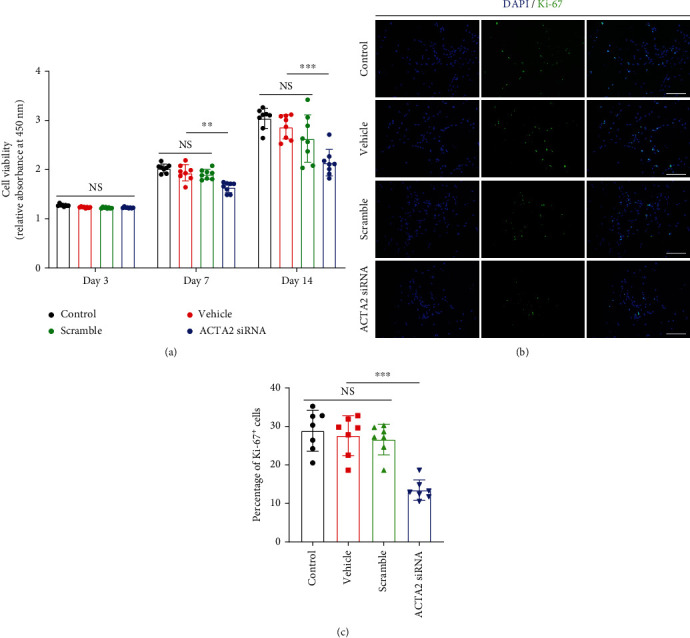
ACTA2 downregulation attenuated NSC proliferation. (a) CCK8 assays exhibiting cell viability in various groups on days 3, 7, and 14. (b) Immunostaining images depicting the percentage of Ki-67^+^ cells in each group on day 7. Cell nuclei were counterstained with DAPI in blue. Scale bar: 50 *μ*m. (c) Bar chart summarizing the percentage of Ki-67^+^ cells in each group. ^∗∗∗^*p* < 0.001; NS: no statistical difference.

To assess the role of ACTA2 in NSC expansion, primary NSC were obtained from E14.5 C57BL/6 mice, and ACTA2 was downregulated using ACTA2 siRNA, as previously described [16]. The results collected from CCK8 assays indicated that ACTA2 downregulation prominently decreased the cell viability on days 7 and 14 (Figure 2(a)), while no significant difference was observed on day 3 (Figure 2(a)). Meanwhile, the immunostaining of Ki-67 was applied to certify the results obtained from CCK8. The results depicted that the percentage of Ki-67^+^ cells in group ACTA2 siRNA was evidently reduced compared to the other three groups (control, vehicle, and scramble) (Figures 2(b) and 2(c)).

### 3.3. ACTA2 Downregulation Attenuated NSC Direction into the Neuron, While Directing NSC into Astrocytes and Oligodendrocytes

**Figure 3:**
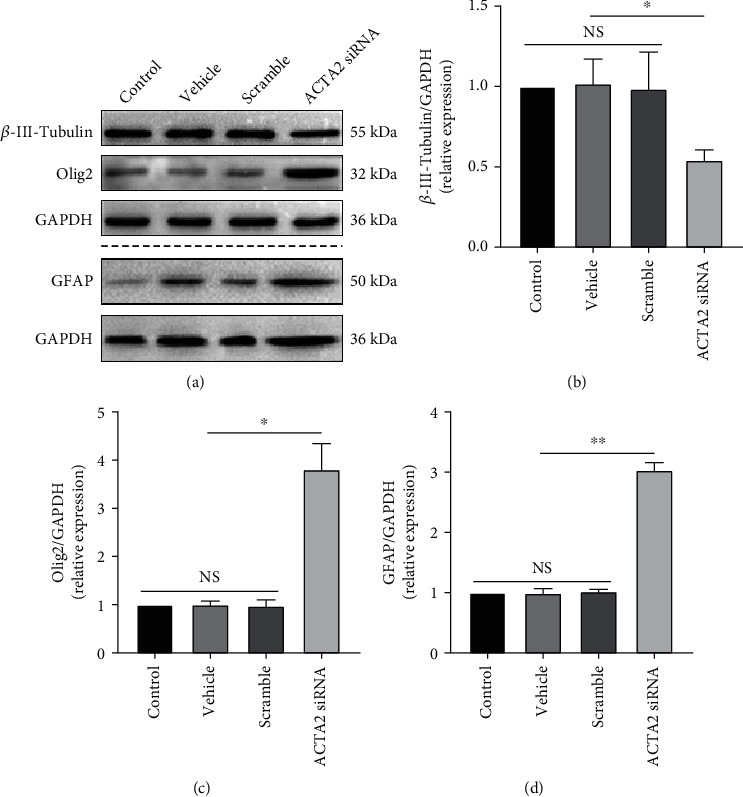
ACTA2 downregulation inhibited NSC differentiation into neurons, while directing NSC into oligodendrocytes and astrocytes. (a) Immunoblotting bands demonstrating the expression of *β*-III-Tubulin, Olig2, and GFAP in each group on day 10. GAPDH was used as a loading control. (b) Semiquantitative analysis of *β*-III-Tubulin expression from (a). (C) Semi-quantitation of Olig2 expression from (a). (d) Semi-quantitative analysis of GFAP expression from (a). ^∗^*p* < 0.05, ^∗∗^*p* < 0.01, and ^∗∗∗^*p* < 0.001; NS: no statistical difference.

**Figure 4:**
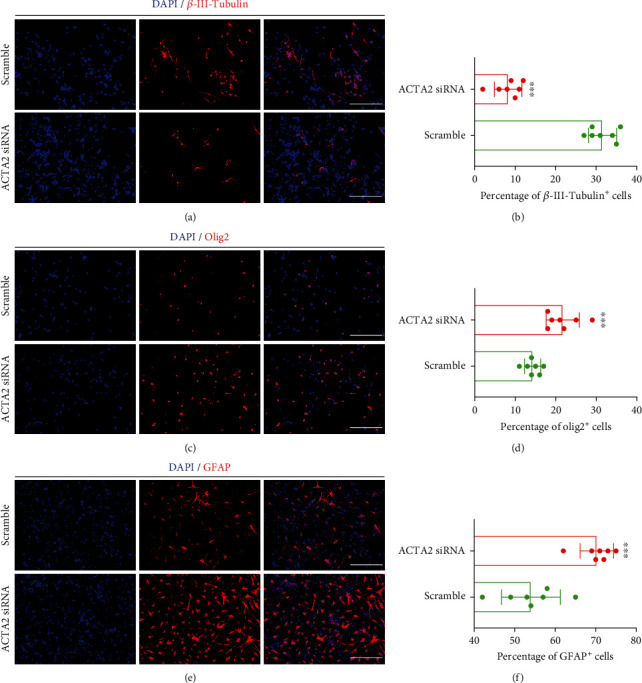
ACTA2 downregulation attenuated NSC differentiation into neurons, while facilitating NSC differentiation into astrocytes and oligodendrocytes. (a) Representative immunostaining images depicting the percentage of *β*-III-Tubulin^+^ cells in each group on day 10. Cell nuclei were counterstained with DAPI in blue. Scale bar: 50 *μ*m. (b) Statistical analysis illustrating the percentage of *β*-III-Tubulin^+^ cells from (a). ^∗∗∗^*p* < 0.001; NS: no statistical difference. (c) Typical immunostaining images illustrating the percent of Olig2^+^ cells in each group on day 10. Cell nuclei were counterstained with DAPI in blue. Scale bar: 50 *μ*m. (d) Bar graph demonstrating the percentage of Olig2^+^ cells from (a). ^∗∗∗^*p* < 0.001; NS: no statistical difference. (e) Representative immunostaining images demonstrating the percent of GFAP^+^ cells in each group on day 10. Cell nuclei were counterstained with DAPI in blue. Scale bar: 50 *μ*m. (f) Bar chart summarizing the percentage of GFAP^+^ cells from (a). ^∗∗∗^*p* < 0.001; NS: no statistical difference.

To certify the effect of ACTA2 downregulation on NSC differentiation, immunoblotting was firstly used to determine the expression of three main neural cell markers derived from NSC (*β*-III-Tubulin for neurons, Olig2 for oligodendrocytes, and GFAP for astrocyte). The bands depicted that the expression of *β*-III-Tubulin was indeed decreased (Figures 3(a) and 3(b)), while the expression of Olig2 and GFAP was remarkably elevated (Figures 3(a), 3(c), and 3(d)), demonstrating that ACTA2 downregulation promoted NSC differentiation into glial lineages (oligodendrocyte and astrocyte), instead of neuronal lineage (neuron). Furthermore, the immunostaining images showed that the percentage of *β*-III-Tubulin^+^ cells was markedly reduced in group ACTA2 siRNA, in comparison with group scramble (Figures 4(a) and 4(b)). Meanwhile, the portion of Olig2^+^ cells was significantly augmented in group ACTA2 siRNA, compared with group scramble (Figures 4(c) and 4(d)). Coincidentally, ACTA2 downregulation profoundly boosted the ratio of GFAP^+^ cells in group ACTA2 siRNA than group scramble (Figures 4(e) and 4(f)). Together, these results revealed that ACTA2 downregulation inhibited NSC differentiation into the neurons, while directing NSC differentiation into glial lineages including astrocytes and oligodendrocytes.

### 3.4. The Canonical Wnt/*β*-Catenin Signaling Pathway Was Involved in the Effect of ACTA2 Downregulation on the Proliferation and Differentiation of NSC

**Figure 5:**
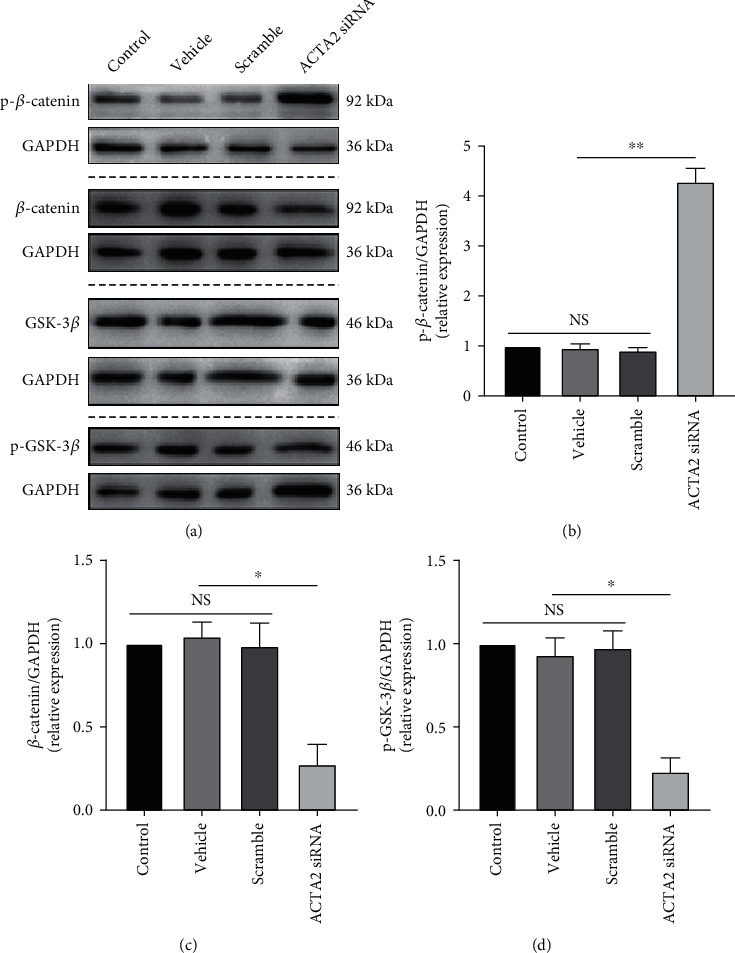
ACTA2 downregulation promoted *β*-catenin accumulation through inactivating GSK-3*β*. (a) Immunoblotting bands displaying the expression of p-*β*-catenin, *β*-catenin, and GSK-3*β* and p-GSK-3*β* in each group on day 10. GAPDH was used as an internal control. (b) Semiquantitative analysis of p-*β*-catenin expression from (a). (c) Semiquantitation of *β*-catenin expression from (a). (d) Semiquantitative analysis of p-GSK-3*β* expression from (a). ^∗^*p* < 0.05 and^∗∗^*p* < 0.01; NS: no statistical difference.

To unveil the possible mechanism underpinning the effect of ACTA2 downregulation on the proliferation and differentiation of NSC, we speculated that Wnt/*β*-catenin signaling pathway might be associated with this effect due to the pivotal role of Wnt/*β*-catenin signaling pathway in regulating proliferation and differentiation of NSC [22]. Initially, immunoblotting was used to examine the expression of p-*β*-catenin and *β*-catenin. The bands displayed that the expression of p-*β*-catenin was prominently increased with ACTA2 downregulation (Figures 5(a) and 5(b)). Meanwhile, ACTA2 reduction promoted *β*-catenin accumulation (Figures 5(a) and 5(c)). Given that GSK-3 negatively mediates *β*-catenin degradation and its phosphorylation induces *β*-catenin deposition [22, 25, 28], the expression of GSK-3*β* and p-GSK-3*β* was assessed by immunoblotting. The results indicated that the expression of GSK-3*β* showed no prominent difference among these four groups (Figure 5(a)), while the expression of p-GSK-3*β* in group ACTA2 siRNA was significantly reduced, compared to the other three groups (Figures 5(a) and 5(d)), indicating that *β*-catenin reduction resulting from ACTA2 downregulation might mediate the proliferation and fate determination of NSC.

**Figure 6:**
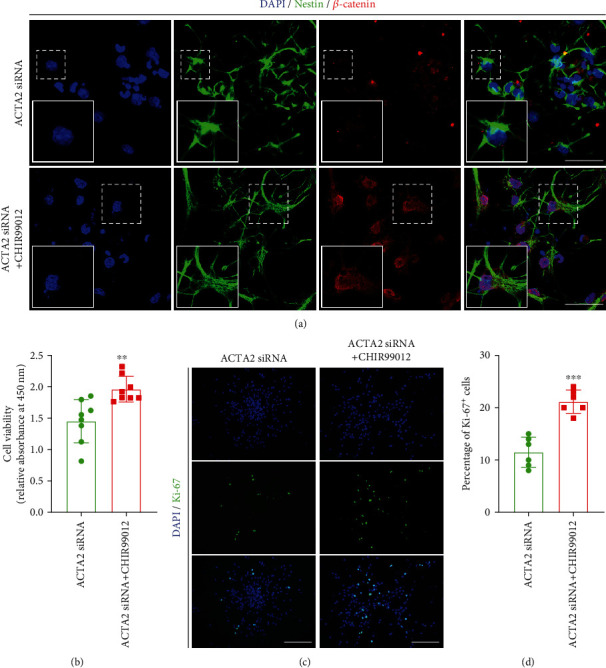
CHIR99012 facilitated the proliferation of NSC via increasing *β*-catenin deposition. (a) Representative immunostaining images representing the expression of *β*-catenin in NSC with addition of CHIR99012 on day 7. Cell nuclei were counterstained with DAPI in blue. Scale bar: 20 *μ*m. (b) CCK8 assays exhibiting the cell viability between group ACTA2 siRNA and group ACTA2 siRNA+CHIR99012 on day 7. ^∗∗^*p* < 0.01. (c) Immunostaining images indicating the percentage of Ki-67^+^ cells in each group on day 7. Cell nuclei were counterstained with DAPI in blue. Scale bar: 50 *μ*m. (d) Bar chart summarizing the percent of Ki-67^+^ cells in each group. ^∗∗∗^*p* < 0.001.

Based on the above results, CHIR99012, a GSK-3 inhibitor that activates Wnt signaling [22], was used to learn the role of the canonical Wnt/*β*-catenin signaling pathway in the effects of ACTA2 downregulation on NSC proliferation. Primarily, the results indicated that the fluorescence intensity of *β*-catenin in group ACTA2+CHIR99012 was substantially increased with administration of 3 *μ*M CHIR99012, compared to group ACTA2 siRNA (Figure 6(a)). Next, the CCK8 assays presented that the cell viability was remarkably increased with the addition of 3 *μ*M CHIR99012 on day 7 (Figure 6(b)). Then, the immunostaining of Ki-67 was performed to investigate the role of CHIR99012 in regulating NSC proliferation after ACTA2 downregulation using the immunostaining method. The immunostaining images depicted that the proportion of Ki-67^+^ cells was markedly increased with administration of 3 *μ*M CHIR99012 (Figures 6(c) and 6(d)). Collectively, these results illustrated that 3 *μ*M CHIR99012 could partially abrogate the inhibitory effect of ACTA2 reduction on NSC proliferation.

**Figure 7:**
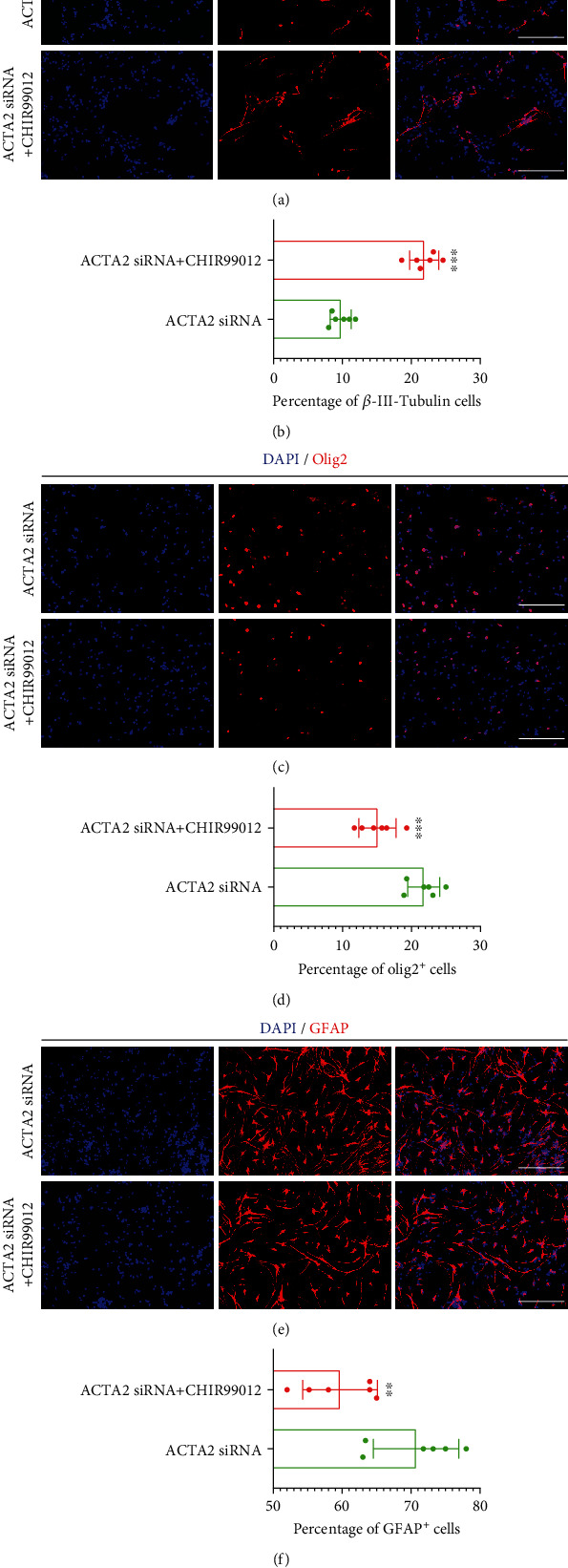
CHIR99012 reinforced the differentiation of NSC into neurons, while suppressed NSC differentiation into oligodendrocytes and astrocytes. (a) Representative immunostaining images illustrating the percentage of *β*-III-Tubulin^+^ cells with the addition of CHIR99012. Cell nuclei were counterstained with DAPI in blue. Scale bar: 50 *μ*m. (b) Statistical analysis demonstrating the percentage of *β*-III-Tubulin^+^ cells from (a). ^∗∗∗^*p* < 0.001. (c) Typical immunostaining images depicting the percentage of Olig2^+^ cells with administration of CHIR99012. Cell nuclei were counterstained with DAPI in blue. Scale bar: 50 *μ*m. (d) Bar graph demonstrating the percentage of Olig2^+^ cells from (c). ^∗∗∗^*p* < 0.001. (e) Representative immunostaining images depicting the percentage of GFAP^+^ cells with treatment of CHIR99012. Cell nuclei were counterstained with DAPI in blue. Scale bar: 50 *μ*m. (f) Bar chart summarizing the percentage of GFAP^+^ cells from (e). ^∗∗^*p* < 0.01.

In addition, immunostaining was performed to explore the effect of CHIR99012 on NSC differentiation. The immunostaining images illustrated that the reduced percentage of *β*-III-Tubulin^+^ cells induced by ACTA2 downregulation was partially reversed with administration of 3 *μ*M CHIR99012 (Figures 7(a) and 7(b)). Subsequently, the results recapitulated that the increased ratio of Olig2^+^ cells was dramatically decreased in group ACTA2+CHIR99012, compared to group ACTA2 (Figures 7(c) and 7(d)). Thenceforth, the increased percentage of GFAP^+^ cells caused by ACTA2 downregulation was substantially diminished with the treatment of 3 *μ*M CHIR99012 (Figures 7(e) and 7(f)). Together, these results suggested that administration of CHIR99012 held the capacity of partly eliminating the effect that ACTA2 downregulation directed NSC differentiation into glial lineages including astrocytes and oligodendrocytes and inhibited NSC differentiation into neurons.

## 4. Discussion

In the present study, primary NSC were collected from mice and the expression of ACTA2 was downregulated using ACTA2 siRNA. Thereafter, the effect of ACTA2 downregulation on the proliferation and differentiation of NSC was investigated. The results revealed that ACTA2 downregulation attenuated NSC proliferation and differentiation into neurons derived from NSC, while directing NSC into astrocytes and oligodendrocytes. During this process, the expression level of *β*-catenin inversely correlated with the expression of p-*β*-catenin ascribing to the decreased p-GSK-3*β* expression. Meanwhile, administration of CHIR99012, a GSK-3 inhibitor, partially abrogated the effect that ACTA2 downregulation inhibited the NSC proliferation and promoted them differentiation into astrocytes and oligodendrocytes.

GSK-3*β*, a versatile serine threonine kinase, has been identified to play a pivotal role in mediating the proliferation and differentiation of NSC during embryonic development and neurological diseases [28, 29]. The activation of GSK-3*β* in nonphosphorylated state negatively results in active *β*-catenin degradation to suppress proliferation and differentiation into neuronal lineage [29], which is in line with our results that ACTA2 downregulation leads to active *β*-catenin reduction. Oppositely, the expression of active *β*-catenin is increased with administration of CHIR99012, which maintains GSK-3*β* in a phosphorylated condition to activate Wnt/*β*-catenin pathway [25]. Given that the rescued effect is partial, some other signaling pathways might participate in this regulation loop. A previous study has demonstrated that Sulforaphene (SF), one of the main isothiocyanates isolated from a Chinese herb Raphani Semen, holds the ability of activating PI3K/AKT signaling pathway to enhance p-GSK-3*β* deposition, thereafter exerting neuroprotective effects in Alzheimer's disease (AD) [30]. Meanwhile, research shows that Acylglycerol kinase (AGK), a newly discovered lipid kinase, could increase the nuclear accumulation of *β*-catenin by activating the PI3K/AKT/GSK-3*β* signaling pathway in renal cell carcinoma (RCC) that further potentiates the activity of TCF/LEF transcription factor [31]. These clues imply that PI3K/AKT signaling pathway participates in the process of ACTA2 downregulation mediating the proliferation and differentiation of NSC, which opens a new research focus for our future investigation.

ACTA2 downregulation manipulates actin filament polymerization to influence the characteristics of NSC, including proliferation, migration, and differentiation. Our previous work recapitulates that ACTA2 downregulation mediates actin filament polymerization through decreasing Rac1 and elevating RhoA to inhibit NSC migration and cytoskeleton rearrangement [16]. Here, our study pays the main attention to the proliferation and differentiation of NSC after ACTA2 downregulation by ACTA2 siRNA, which provides evidence for ACTA2 in regulating the function of NSC. The results demonstrate that ACTA2 downregulation attenuates NSC proliferation, which is consistent with previous reports that ACTA2 reduction suppresses cell proliferation including fibroblasts [32], A549, and MRC-5 cell lines [33]. Meanwhile, the results substantiated that ACTA2 downregulation promoted NSC differentiation into astrocytes and oligodendrocytes, while suppressing NSC direction into neurons. The reason for this phenomenon might be ascribed to cytoskeleton rearrangement as cytoskeletal rearrangement augments the differentiation of mesenchymal stem cells (MSCs) into neurogenic subtype [34]. Combined with our previous work, this study serves as supplementary evidence for elucidating the role of ACTA2 in controlling the expansion and fate determination of NSC, which would provide a locus for gene modification in cell replacement treatment.

The advantages of cell-based therapy for NSC after neurological disorders include the following: (1) cell replacement for the injured neurovascular network; (2) immunomodulation through inhibiting the release of proinflammatory factors; (3) enhancing neurotrophic factors deposition to maintain the survival of damaged neural cells; and (4) improving the local microenvironment after injury [4, 35, 36]. However, the insufficient number of NSC originated from SVZ and migrating toward the infarct core restricts the regenerative capacity of NSC after ischemic stroke [5, 15]. Furthermore, investigations have shown that most of the newborn NSC differentiate into astrocytes to form glial scars after central neuropathy [13–15]. Therefore, several approaches have been employed to enhance the neurorestorative ability of NSC including increasing the potential of proliferation and migration of NSC and promoting NSC differentiation into functional neurons. Gene modification is one of the methods for exerting neurorestorative effects associated with cell replacement therapy using exogenous NSC. Here, our research offers a feasible locus for gene modification that ACTA2 overexpression might hold the capacity of facilitating proliferation, migration, and proper differentiation of exogenous NSC when transplanted after central neuropathy. Hence, the research emphasis for our future work is to introduce a model associated with central neuropathy and to assess the therapeutic effect of modified NSC implantation on attenuating the pathological deterioration and facilitating functional recovery.

## 5. Conclusions

In sum, the present study indicates that ACTA2 downregulation inhibits NSC proliferation and differentiation into neurons, while directing NSC into astrocytes and oligodendrocytes through inactivating of the canonical Wnt/*β*-catenin pathway, which provides a feasible intervention target for promoting NSC proliferation and proper differentiation.

## Data Availability

The data used to support the findings of this study are available from the corresponding author upon reasonable request.
